# Relooking at the Roles of Translanguaging in English as a Foreign Language Classes for Multilingual Learners: Practices and Implications

**DOI:** 10.3389/fpsyg.2022.850649

**Published:** 2022-06-07

**Authors:** Ping Wang

**Affiliations:** School of Foreign Languages, Jiangsu University of Science and Technology, Zhenjiang, China

**Keywords:** EFL, Neidi class, translanguaging pedagogy, classroom practices, Xinjiang

## Abstract

The use of translanguaging pedagogy rather than favoring English-only teaching put those who hold monolingual ideology in foreign language pedagogy. A study of English as a foreign language (EFL) for multilingual students showed that embracing translanguaging appeared compelling that it emerged in Neidi Xinjiang Classes (NXC) in China. After reviewing the concepts of translanguaging, this writing presents the translanguaging theory and translanguaging pedagogy. Guided by translanguaging lenses and stances, a longitudinal case study was carried out to look into the EFL pedagogy in NXC. The focal data was heavily drawn from reflective interviews and classroom observations. The data analyses were particularly paid on the triangulation of data with the interviews and classroom observational data. The findings indicate the students made full use of the affordances to do translanguaging practices while a few EFL teachers with the monolingual ideology still violated English-only constraints against the background of the multilingual turn. Therefore, the conclusion builds on dynamic multilingual stances to identify the significance of enabling the presence of translanguaging pedagogy.

## Introduction

English as a foreign language (EFL) has been trapped in the English-only EFL pedagogy for the last three decades (Kramsch, 2014; Hall, 2018; Cenoz and Gorter, 2019), and the learners’ multilingual background in EFL pedagogy was neglected, being in an all-or-nothing state (Cenoz and Gorter, 2014; Hall, 2016a,b, 2018; Li, 2018). A substantive issue was raised: how to teach multilingual learners when translanguaging pedagogy was introduced.

Otheguy et al. (2015, p. 281) explain that translanguaging uses the bilingual learners’ entire linguistic repertoire “without regard for watchful adherence to the socially and politically defined boundaries of named languages.” This definition of translanguaging refers to teachers taking into account bilingual learners’ unitary linguistic system and managing to offer learners opportunities to leverage their full linguistic repertoires instead of considering using a particular named language. Translanguaging in EFL pedagogy specifically has been described as the ability of multilingual learners to utilize their entire linguistic repertoires to reach the goal of English learning (García and Li, 2014).

Neidi Xinjiang class (NXC) refers to selecting outstanding junior high school graduates in Xinjiang Uyghur Autonomous Region to study in high schools in China’s central and eastern developed regions. The reason behind establishing NXC is that the economic and educational advantages of the developed areas could be used to improve the quality of education for students of all ethnic groups as a cultural and cross-regional cooperative education model. As an ethnic minority education policy with national strategic significance, since its establishment in 2000, the NXC has continuously expanded the enrollment scale, enormously satisfying the urgent needs of the school-age youths of all ethnic groups in Xinjiang to receive 4 years of the NXC education. The NXC has achieved outstanding results, but some issues related to the EFL pedagogy have inevitably arisen.

Interestingly, teachers decide what is most relevant (Hall and Cook, 2014) and appropriate to adapt the translanguaging theory to synergistic relation with the Neidi NXC program, which is worth studying at length. This writing mainly focuses on identifying the mindset of EFL teachers and students in EFL contexts towards including translanguaging in their classrooms and how and when principled translanguaging occurs in NXC classrooms to fulfill EFL pedagogical aims. This research hoped to follow the broad range of ideas identified in translanguaging perspectives and make some new contributions to the related field.

## Literature Review

Canagarajah (2011, p. 401) defines translanguaging as “the ability of multilingual speakers to shuttle between languages, treating the diverse languages that form their repertoire as an integrated system.” García and Li (2014) contest the viewpoint of Canagarajah and argue that it is not merely a shuttle between languages but denotes speakers’ construction and use of original and complex interrelated discursive practices that cannot be easily assigned to one or another traditional definition of a language, but that makeup of speakers’ complete language repertoire. The extant literature shows that translanguaging can be a new insight into understanding bi/multilingual practices. García (2009, p. 45) puts that translanguaging is a bilingual’s “multiple discursive practices in which bilinguals engage to make sense of their bilingual worlds.” Li (2018, p. 23) argues that “translanguaging is not a thing in itself,” and it is not an object to describe, and it is a lens to capture the hybrid dynamic discursive practices, which endorses a flexible view of named languages (Durán and Palmer, 2014). This study used definition of translanguaging pedagogy of García and Li (2014) related to translingual practices of Canagarajah (2013). Language teachers use all students’ repertoires to support the bi/multilingual students’ learning of English.

The term translanguaging, in this manuscript, is often discussed in the way of pedagogical translanguaging. Pedagogical translanguaging refers to using translanguaging practice for pedagogical purposes (Cenoz, 2017). It is a pedagogical approach in bi/multilingual education, effective in meaning-making/negotiation by engaging their unitary but multiple repertoires. The significance of the unitary repertoire lies in the bi/multilingual speakers’ linguistic resources being kept as a whole, and they select the right help from the entire repertoire to meet their communicative needs. According to Li (2018), their repertoires also contain multimodal resources integrated with linguistic resources. As a pedagogical lens, it, to some extent, validate the bi/multilingual learners’ languages and cultures in school contexts through teachers purposefully counteracting monolingual ideology and creating an ideal democratic translanguaging space for teachers and students to perform dynamic, fluid, and creative translanguaging practice to “co-create knowledge” (García et al., 2017) through enriching their multilingual competence (Cook, 2010). This lens provides concrete foundations to reexamine the roles of teachers and the parts of students and relook at the goals of pedagogy and the process of classroom learning and teaching in the multilingual era.

In monolingual views of language teaching, translanguaging in the EFL classroom will statistically minimize and even diminish the amount of exposure to practicing a new language. The students who mix their linguistic repertoires often discourage their teachers because overuse hinders the latest language acquisition process. This is the well-known reason scholars with a translanguaging-as-an issue ideology have argued. According to Palmer (2011) and Sayer (2013), teachers’ decision-making towards translanguaging largely depends on their language ideology, which is “sets of beliefs about language articulated by users as rationalization or justification of perceived language structure and use” (Silverstein, 1979, p. 193). EFL teachers’ language ideologies significantly influence (often encouraging and limiting) the students’ translanguaging practices. Teachers’ monolingual ideology may cut learners off their entire repertoire (García and Li, 2014). Reexamining the language ideology deeply entrenched means shaping their stances toward the heteroglossic perspective translanguaging.

Judicious use of translanguaging can be the last resort. According to Williams et al. (1996, p. 9), “purposeful translanguaging is needed.” Echoing Williams et al., there have been many calls for research into differing bi/multilingual learners’ languaging competence, resulting in the judicious use of learners’ resources stored in their linguistic repertoires. García et al. (2017) mentioned three framing concepts of translanguaging pedagogy: stance, design, and shifts. Translanguaging stance as critical notions focuses on valuing bi/multilingual learners’ fill linguistic repertoires as learning resources. Translanguaging design stresses that purposive translanguaging strategies should be designed before being implemented. Translanguaging shifts are the expected results of translanguaging pedagogical practices, in which teachers entirely employ explicit strategies to use all the learners’ linguistic resources. The three perceptions are accommodated within a translanguaging stance/lens of leveraging the learners’ linguistic repertoires to acquire English (Conteh, 2018).

Although the existing translanguaging theory can provide a more rational analysis and interpretation of the complexity of EFL, it has not been successful in attracting extensive attention from the NXC EFL community. That is to say; there are very few systematic studies on related topics compared to the large number of studies conducted in the United States, the United Kingdom, Spain, Canada, Iceland, Norway, Italy, Singapore, Malaysia, etc. In other words, although a large number of empirical researches and theoretical modeling studies have been carried out on the translanguaging theory in various educational contexts across the world, the empirical translanguaging research on the NXC EFL teaching is still rare.

## Materials and Methods

This study frames the NXC EFL classroom as a community of translanguaging practice where students’ and teachers’ linguistic resources serve as practical tools that shape the community, of course, and in turn, the community of practice shapes how the tools are or are not used.

### Research Questions

This qualitative study examines how school teachers and students participated in the translanguaging EFL pedagogy over an academic year. Specifically, it addresses the following three research questions.

1. To what extent is translanguaging pedagogy engaged in the NXC EFL classrooms?
2. How do teachers’ language beliefs impact their teaching strategies and the students’ ability to use translanguaging to support their new language learning?

### Research Design

In the current study, a case study (Yin, 2018) was employed regarding the self-identified translanguaging pedagogy of the learners studying within an NXC context to gain an initial understanding of the translanguaging phenomena in EFL teaching for the NXC. “The case study is not a specific technique but a method of collecting and organizing data to maximize our understanding of the unitary character of the social being or object studied. In many ways, it is the ultimate qualitative method focusing on ‘Particular One’“(Dörnyei, 2007, p. 152).

### Context

In 1984, the Chinese government put forward the slogan “Intellectual aid to Tibet,” requiring the provinces and cities in mainland China to prepare for establishing Tibetan middle schools and open inland Tibet classes. The success of the *Neidi Tibet Classes* in 2000 paved the way for the opening of the *Neidi Xinjiang Classes*. Both the *Neidi Tibet Class*es and the *Neidi Xinjiang Classes* aim to make full use of the high-quality educational resources of the developed provinces and municipalities in the mainland to cultivate young talents for ethnic minorities in Tibet and Xinjiang.

The school with Neidi Xinjiang Classes for the fieldwork in this study is located in East China and is an anonymously A Middle School. The school has a long history, and famous people have large numbers. This school opened *Neidi Xinjiang Classes* in the fall of 2006. After the selection examination for *Neidi Classes* held in Xinjiang Uygur Autonomous Region, it opened the first four *Neidi Xinjiang Classes* with 125 students. Enrollment targets include five ethnic groups from southern Xinjiang, including Uyghur, Kazakh, Kirgiz, Tajik, and Mongolian. The school has successively won the honorary titles for the outstanding performance of *Neidi Xinjiang Classes*.

The *Neidi Xinjiang Classes* of this school require 1 year of preparatory courses, which mainly connect to the practices of high schools in Xinjiang. After that, it is divided into 3 years of high school. Thus, the *Neidi Xinjiang Classes* have four grades, each grade having four classes. The *Neidi Xinjiang Classes* have an independent teaching building, canteen, and dormitory and are equipped with Xinjiang full-time counselors to be responsible for students’ daily management and psychological adjustment and organize some after-school activities. After graduating from the four-year high school, students will take the local college entrance examination.

### Participating Teachers

Three focal teachers were purposively selected from the larger cohort of the teachers who participated in the study (see Table 1). Those sets had different views towards orchestrating the entire linguistic repertoires for learning a new language. The three were treated as an individual units, respectively, who were observed and interviewed for their real-class translanguaging practices.

**Table 1:** Participants: EFL teachers.

Name (Pseudo)	Gender	L1	L2	L3	Education	Teaching years
Teacher A	Male	Chinese	English	French	MA in TEFL	19
Teacher B	Female	Chinese	English	Japanese	MA in TEFL	10
Teacher C	Female	Chinese	English	French	MA in TEFL	6
Teacher D	Female	Chinese	English	German	MA in TEFL	5

All the participant teachers held MA in EFL teaching certificates, and they are typical Chinese and English bilinguals. They did not have the same languages as their students. They mainly communicated with students in Chinese or English since the teachers generally cannot speak Uyghur or Kazak beyond the word “ياخشىمۇسىز، ئامانمۇسىز,” which means hello in Uyghur. The teacher participants who agreed to participate in the project had the opportunity to inquire about their involvement and were offered a consent form that they could sign.

At the beginning of the semester across school years, the EFL teachers of NXC must attend seven afternoons of training sessions, enabling them to align the pedagogical beliefs to actual translanguaging pedagogy. The four primary identified purposes of the training are as follows:

1. Engagement aspects: gaining bi/multilingual learners’ attention; engaging them with strategic use of translanguaging pedagogical techniques.
2. Teacher awareness: be aware of the benefits of employing the students’ full linguistic repertoires, reflecting upon what they genuinely believe by relooking beyond their classroom practices and linking their reflective accounts to leveraging their entire linguistic repertoires.
3. Translanguaging theoretical and practical perspectives: Introducing the term translanguaging, drawing upon translanguaging publications from Garcia, Li, and other scholars, and knowing the translanguaging theory through comprehending the publications; guiding them to value students’ full linguistic repertoires; giving them agency in a sense/meaning-making; providing them the sampled activities of negotiating meaning, cooperative learning, and removing the learning barrier through promoting dynamic bi/multilingualism or plurilingualism.
4. Required skills for applying translanguaging pedagogical strategies to foster the learners’ pluriliteracies.

After training, most of them knew how to use translanguaging theory in EFL pedagogy, and they had mastered how to use their multilingualism to communicate across language barriers in the NXC setting.

### Participating Students

One hundred ninety-seven students were involved in this investigation. The NXC cohort, on average, had studied English for more than 5–7 years, which indicated that they are likely to have developed at least basic English proficiency. Another feature of the participants was that they studied Chinese as their L2, which had also been used as their instruction language since they started schooling. Their L1s are mostly Uyghur or Kazak, commonly spoken in their daily communications among students. The class size ranged between 37 and 45 students.

### Data Collection

Three data sets were collected during one academic year at the case school. Eight-week classroom observations were conducted, which extended up to 16 h. This was supplemented with observers’ field notes to record incidents regarding the teacher-student interactions.

The classroom observation data, which took place within the autumn term, were first collected. During the observations, we used Instructional Environment Profile for observing 18 features of the organization of instruction as well as languaging practices carried out by teachers and students; the student Functional Proficiency profile for three areas: students engagement, task completion, and description; and the Description of Instruction Practice profile for teachers’ encouraging students’ attention, allowing students’ interactive translanguaging, exhibiting multicultural identities, and emphasizing the meaning-making rather than different named language structures.

During the semi-structured interviews that delved into the teachers’ current beliefs and past learning experiences, we asked them questions about their perceptions of (1) how the teachers’ ideologies influence/impact the translanguaging pedagogy concerning their reactions to translanguaging theory, (2) students’ translanguaging enactions and their classroom language policy, and (3) the reasons for their translanguaging practice.

The third data collection component was the semi-structured interview (see Table 2). The students’ interview content focused on using other languages rather than English. One chief goal of the students’ interviews was to determine the reactions to their teachers’ translanguaging in the EFL classroom.

**Table 2:** Data sources.

Instruments	Number	Duration	Average	Format
Semi-structure interviews with teachers	4	4 h 39 m	1 h	Audio-recorded
Semi-structure interviews with students	9	4.6 h 15 m	0.5 h	Audio-recorded
Classroom observations and fieldnotes	8	8 months	2 days a month	Audio-recorded

As non-participatory researchers, once the NXC started, we kept a close eye on the pedagogical progress. We sat in during the classes during the classroom observation, took field notes, and managed to limit our presence by informing teachers ahead of time that we were there for observation purposes only instead of strictly evaluating the teacher. Therefore, the organic data collected was more likely to represent the teachers’ regular practices.

### Data Analysis

To ensure data analysis, we conducted five rounds of inductive and deductive coding and axial coding (Gee and Hayes, 2011) to handle the multiple data collected, with the research questions born in mind. The first round of data analysis focused on analyzing the transcriptions from the 12 interviews (four with teachers and eight with students) and the translanguaging interactions during the classes observed to determine the affordances or constraints of translanguaging in students learning. Such factors as the physical classroom space, the participants’ backgrounds, and the contextual elements were witnessed to see if all these factors would impact the EFL pedagogy. The transcriptions were axially coded in the second round to understand the research questions in-depth. In the third round, iterative data analyses were used to identify the categories of translanguaging constellation and the means of translanguaging pedagogical interaction: (1) English; (2) Chinese; (3) Uyghur/Kazak; (4) English and Uyghur/Kazak; (5) English, Chinese and Uyghur/Kazak; and (6) Chinese and Uyghur/Kazak. In the fourth round, the possible relationships between the ideologies of teachers and students and the factors influencing students’ use of translanguaging practices in class were addressed by grouping the pertinent quotes from the interviews with both teachers and students. In the last round, the data from the observations and the two streams of interviews were interactively analyzed to guarantee a triangulating examination to avoid biased interpretations (Marchel, 2007, p. 1) and to create in-depth descriptions that integrated multiple perspectives into the accounts and painted a fuller picture of the phenomenon of translanguaging revealed in the NXC EFL pedagogical context. To ensure the authenticity of data, member checks were conducted to compare thoughts and inductively code upon multiple sources (Maxwell, 2013), and peer debriefing was employed to enhance the trustworthiness of the answers to the research questions (Lincoln and Guba, 1985).

Before obtaining access to the research site, researchers have decided to use pseudonyms for the participating schools, classes, and all the students and teachers voluntarily involved in this research, and the participants would be kept confidential.

## Results

The findings presented below were drawn from the classroom observations and interviews with teachers and students.

### Translanguaging Phenomena

The translanguaging phenomena that we have seen as a classroom observers can be briefly divided into three types: teacher-led, student-led and blended.

Firstly, the NXC EFL teacher habitually provided Chinese (L2 for students) equivalents for crucial terms, demonstrating a pre-disposition to translanguaging to support learning. Sometimes it seemed that the L2 (Chinese) provision was a “simple” translation to strengthen L3 (English) learning. Secondly, the minority students themselves considerably influenced and determined flexible language arrangements as trilingual learners in a classroom, needing to communicate their conceptual understanding or misunderstanding immediately and efficiently. They worked independently and usually completed the activity by co-constructing meaning and mediating interpretation in multimedia-assisted classroom learning contexts. They also gathered information from the Internet in L3 and/or L2, or L1 outside of the classroom, discussed the content in L3 and/or L2, or L2 and/or L1, and completed the written work in L3, a target language. Thirdly, some EFL teachers’ translanguaging was fluid when accepting the students’ choice of language codes or returning to the original code after switching. We frequently found that an NXC EFL teacher would code-switch back and forth between English and Chinese while using explanation and repetition to help students understand his instruction for a group activity in which students needed to make their own choices.

### Creating a Translanguaging Space and Leveraging Learners’ Entire Linguistic Repertoires

Most teachers tried their best to create a translanguaging space, where teachers and students can be offered the translanguaging moments to orchestrate their entire repertoires to facilitate classroom EFL learning and teaching. The following is an example observed in classes.

Teacher A (*pointing to a new word on the whiteboard, on which is written* status quo) said the phrase “status quo” (现状/xianzhuang in Chinese) commonly refers to “*the situation as it is now, or as it was before a recent change: new challenges to the status quo include terrorism and the spread of nuclear weapons*.” Other examples are:

1. *Frederick the Great would have been happy to maintain the status quo.*
2. *to preserve*
3. *challenge the status quo*

Now work in a group of four or five, and discuss the meaning of “status quo,” the teacher continued.

The students (with different language backgrounds: two Uyghur and three Kazak): started the discussion by using Chinese on two levels: first, understand the teacher’s requirement, and then translate the English explanation into Chinese to make meaning:

Level one: 老师让我们讨论 status quo的意思。.

Level two: status quo指的是“现在或最近变化之前的状况。老师给出的例子的意思:“人类面临来自挑包括恐怖主义和核武器扩散的现状。”

1. Frederick大帝很乐意维持现状。
2. *to preserve*保持现状/*challenge the status quo*挑战目前的现状

In contrast, the students with the same or similar background started to use Uyghur directly to make meaning: بىرىكمە Status quo_ ھازىرقى ۋەزىيەت ياكى يېقىنقى ئۆزگىرىشلەردىن بۇرۇنقى ئەسلىي ھالەتنى كۆرسىتىدۇ. مۇئەللىم كۆرسەتكەن مىسال: “ئىنسانىيەتنىڭ نۆۋەتتىكى مەۋجۇتلۇقى تېرورىزم ۋە يادرو قوراللىرىنىڭ يامراپ كېتىشىنى ئۆز ئىچىگە ئالغان خىرىسلارغا دۇچ كېلىۋاتىدۇ.” Status quoنىڭ تېخىمۇ كۆپ قوللىنىش ئۇسۇللىرىدىن تۆۋەندىكىلەر بار:

1. Frederick ئېمپىرىيە نۆۋەتتىكى ۋەزىيەتنى ساقلاشنى خالايدۇ.
2. ھازىرقى ۋەزىيەتنى ساقلىماق/نۆۋەتتىكى ۋەزىيەتكە جەڭ ئېلان قىلماق

When the teacher approached close to the Uyghur group, they shifted their discussion to Chinese mixed with some English words: “status quo意思是‘状况’。白板上的例句的意思:‘目前的现状not very good, 有来自恐怖主义和核武器扩散的threat。’….” This articulates a clear position encouraging bilingual pedagogical strategies while discouraging the exclusion of students’ home language in the instruction. Acknowledging students’ organic fluid language practices naturally breaks the static conception of language as an isolated entity with entrenched monolingual bias. Translanguaging can be the vehicle for enriching the learner’s language repertoires.

Such observations reveal that (a) there is no clear-cut boundary between the bilingual students’ languages; (b) the teachers should treat them as bilingual beings; and (c) translanguaging is an effective pedagogy to activate and improve the students’ translanguaging behavior, through which they collaboratively expressed their thoughts, ideas, and experiences. The students naturally used their L1 or L2 or mixed with both, especially when collaborative work. As the group work progressed, they summarized the key points in their L1.

The critical thing is that teachers should identify the crucial moments to use specific resources drawn from the repertoires. For example, when the teachers have identified the possible barriers that the students were to meet, the teachers should actively encourage the learners to do translanguaging practices through which the obstacles could be erased one after another. Thus, it is a must to offer a place for students to articulate their views.

I could quickly identify the bi/trilingual students speaking in their L1 or Chinese, remind them to practice speaking English, and push them slightly. Nevertheless, I have never prevented them from speaking any other language than English in my EFL class. My class has several translation activities, and Google translation is always encouraged, especially for writing tasks. To my enjoyment, my students translanguaged most of the class time, except that a few would like to speak English all the time. Students were free to use their native languages to express themselves in specific cases, such as explaining ethnic culture, understanding grammar, etc.

The majority of the teachers in the NXC contexts have set good examples for creating the translanguaging space. The teacher has recognized the students’ regular action of translanguaging through which the learners can comprehend the new knowledge. Also, teachers were aware of the positive effect of multimedia facilities and made full use of them and the resources in students’ multimodal repertoires to create a translanguaging space to maximize their learning.

As a high experienced EFL teacher in this school, I have had 20 years of teaching, and I engaged my students with multiple resources in class, including various languages, videos, audio, images, and multilingual texts. The classroom activities were usually organized inhomogeneous groups to facilitate their translanguaging practices and fruitful discussion (Teacher C).

As an experienced teacher, he positioned himself as a total proponent of multimodal translanguaging pedagogy. The above transcript also reflects how the teacher strategically implemented the translanguaging pedagogy to leverage the students’ linguistic repertoires.

As mentioned earlier, the pedagogical usefulness of translanguaging is warranty well-acknowledged, which aligns with the multilingual students’ linguistic resources as a cornerstone to promote cooperative communications. The teacher should lead and act in concert with this supporting actor/actress, alongside the learners who take on leading roles in this drama. Only in this way might the translanguaging pedagogy be marked as an approach to learning effectively. Translanguaging can perpetuate the learner’s target language acquisition by teachers not knowing the students’ family language. Thankfully, Chinese (L1 for teachers but L2 for students) could bridge the learning gap, resulting in translanguaging in English grammar acquisition.

### Mixed Attitudes Toward Translanguaging

If teachers and students co-construct translanguaging pedagogy, it is necessary to look at the beliefs that make the pedagogy possible.

### Translanguage as a Problem/Barrier

A group of male Kazak students with relatively low Chinese language skills exemplified their dilemma. Here is an example:

When I started speaking my language, my English teacher, who was incapable of speaking Uyghur, was like, say, in either English or Chinese. One of my roommates told me that her English teacher had informed her that it was impolite for her to talk in Uyghur to her teacher. Some of my peers were in trouble understanding the teacher without whispering to each other in their native languages. They complained that they could hardly remember what they had learned in class. The teacher had expected that all classroom communications should only occur in English, excluding the students' native languages as resources for English learning. Furthermore, I felt sad at the teacher's marginalizing my language while prioritizing English over others, only following hierarchical sequencing of the wording in the same classroom.

Firstly, the students felt unprepared for translanguage in other languages. The fundamental reason is the current English assessment system, which is still monolingual in China. Thus, students expect the English pedagogy could be conducted in English by skillful English teachers. Secondly, the excerpt also reveals that although teachers strategically use pedagogical translanguaging to show their classroom pedagogy, they are always demanded to perform English monolingual models of language assessment. This conflicting status quo appears between the translanguaging pedagogy and monolingual assessment of students’ performances. Thirdly, students might be concerned about the inadequate shrink amount of exposure to English if other languages are used in an EFL setting. This monolingual stupidity sees the other languages as threats to the target language acquisition.

Despite students’ monolingual ideologies, teachers’ language ideologies were closely linked with encouraging or restricting students’ translanguaging practices.

I have never allowed my students to speak any other language except English for two decades. Speaking different languages can be happening in other teachers' classes, not in mine. I consistently believe that other languages would not help, only ruining the purity of the English they have learned. So, quitting leveraging other English language classes is a unique way to make quick progress. That is why I worked as police in class to warn those who sought opportunities to speak different languages in my class. I failed the other languages would flood, which might show my classroom management ability (Teacher A).

The teacher stated that his students were not permitted to use their native languages, and he viewed translanguaging as an issue, for he committed monolingual bias. That is, he was stuck in the English-only stance.

On the flip side, the teacher did not confess it is the multilingual learners’ natural action when spontaneously mediating their learning but regarded students’ language diversity as an issue to rectify rather than a valued asset to leverage, only resulting in forbidding to use of students’ entire linguistic repertoire and generating devastating effects on students’ agencies. Indeed, the teachers failed to embrace translanguaging pedagogy and embed it. This teacher also had a powerful language hierarchical status among named languages.

### Translanguaging as a Natural Process

However, most of the teachers and students in the NXC program embraced a multilingual ideology. They created and maintained a translanguaging space which means situating the shared L1/L2 in EFL classrooms to provide a solid platform for naturally implementing translanguaging pedagogical practices.

Before introducing translanguaging on the campus, I placed students in heterogeneous language groups to avoid them talking in their native languages. This worked pretty well. The noises from using students' other languages were vastly reduced. However, the emerging problem came out – the silent classroom was there! The majority of students would not talk. After I tried translanguaging pedagogy, my students were shocked at the change in their entire linguistic repertoires. Since then, the students' voices can be heard with their spontaneous translanguaging practices – languages were used in a fluid and constantly shifting way, and translanguaging seems to be a typical classroom practice in my class (Teacher D).

We observed that the student’s level of engagement was developed if translanguaging pedagogy was accepted as an ideal pedagogy. More and more teachers gradually welcomed translanguaging to develop the students’ language engagement for sense-making and mean-making/negotiating abstract words, grammatical terms, and texts across languages. The students’ agency was also exercised in small groups, pairs, and role-plays. Students seem to be more involved in translanguaging activities to demonstrate their knowledge and previous experiences.

Teachers’ adoption of a translanguaging lens means recognizing and valuing, and embracing the students’ nuanced voices and ideas instead of marginalizing the learners’ linguistic diversity, which can help end the issues brought by the monolingual ideology of using one language over another. From a reflective stance, it is a sound decision for teachers to confess the students’ multilingual realities through a translanguaging lens by harnessing the learners’ full repertoires in the classroom EFL pedagogy.

In my English class, the teacher operated under L2 and L3. I naturally involved my native language with L2 and L3 in understanding the teaching thoroughly. Sometimes, I picked the language that would match the situation, like translanguaging. I am constantly comfortable when the teacher allows me to synergize my linguistic resources to clarify and think and express myself (beyond specific languages), resulting in a space for exploring the multilingual practice and maximizing the potential role of the classroom translanguaging practices multimodal resources. Such patterns of hybridity can be easily identified among my peers, and I confirm that my peers have experienced translanguaging trials inside or outside the classroom (Teacher C).

This excerpt shows that the teacher who considered translanguaging a natural practice for the multilingual learners allowed the students to have a wild action of translanguaging.

Compared to teaching English through English, students’ L1 can provide a shortcut for giving instruction and explanation, working through assignments, and resolving problems. Not only is translanguaging pedagogy able to smooth the teacher-student interaction, but student–student interactions will be more focused through translanguaging practice. For an individual student, translanguaging as a natural occurrence effectively establishes an interlink between students’ previous knowledge and would-be knowledge. When the teachers share a common language with the students, both would feel mutually understandable for pedagogy.

I felt at ease and natural at teaching through a translanguaging lens. I flexibly utilized translanguaging to teach grammar and vocabulary, while my students more frequently use translanguaging for fulfilling the classroom activities and making/negotiating the meaning of the text (Teacher A).

The following exhibits the in-depth reasoning:

I do not feel there is a space between the three languages because Uyghur, Chinese, and English always seem to overlap, merge and want to come together, embrace each other, and mesh one into others (Teacher B).

The transcript above voiced explicit assumption that permeates EFL pedagogy and can have dire consequences for the EFL teaching innovation and teachers. Through this assumption, students’ multiple linguistic senses can be stimulated to notice, hear, see, feel, and learn, resulting in better memory, understanding, and enhanced learning efficacy. This assumption is based on two intertwined fallacies about the effectiveness of translanguaging pedagogy and its relationship to students’ consistent learning results. It becomes apparent that the students can better their learning inside translanguaging EFL classrooms, which ultimately liberates the learner agency and provides needed resources for students and teachers. The translanguaging pedagogy frees teachers to devote their energy and time to implementing it with their students rather than teaching their own.

Table 3 showed translanguaging purposes from both the perspectives of teachers and students.

**Table 3:** Translanguaging purposes across the eight observations.

Students translanguaging practices	Results of eight classroom observations							
	T1	T2	T3	T4	T5	T6	T7	T8
To help each other by providing/displaying information	*		*	*	*	*	*	*
To use multilingual dictionaries for reaching an understanding	*	*	*	*	*		*	*
To interact and keep rapports	*		*	*	*		*	*
To request and clarify and negotiate information	*		*	*	*		*	*
To affirm/reject information	*	*	*	*	*	*	*	*
**Teachers translanguaging practices**								
To check understanding with elicitation	*	*		*	*	*	*	*
To present and explain lesson contents in a simplified manner	*	*	*	*		*	*	*
To expeditiously explain a vocabulary/grammatical item through translation	*	*		*	*	*	*	*
To manage the class and facilitate task administration	*		*	*	*		*	*
To enhance teacher-student personalized contacts and build a good rapport		*	*	*	*		*	*
To support students’ identity actively	*	*	*		*	*	*	*

*Agreeing with the questionnaire item.

### Teachers’ Translanguaging Practices

The following illustrates how the teachers embraced and incorporated translanguage strategies into the EFL classrooms. These strategies might include: First, teachers setting up translanguaging space and allowing students to leverage all their repertoires; Second, teachers’ pairing students according to their native languages; Third, teachers’ encouraging students to use multilingual dictionaries; Fourth, teachers’ encouraging students to gain help from their peers; Fifth, teachers’ greeting students with students’ L1; Sixth, teachers encouraging the collaborative way of learning (pair work, group work, and teamwork); Seventh, teachers providing students with opportunities of using their native cultural knowledge; Eighth, teachers encouraging students to read in their language.

The time ESFL teachers’ translanguaging was varied across different individual teachers. Teachers spoke only 70% of the time they were observed, and students said 48% of the time they were kept. As for translanguaging practices across four classes, only one teacher used English in more significant proportions than in the other three classes, in which students used English in more significant proportions than the ones in the other three classes. Moreover, for 50% of the class time we observed, students did either pair work or group work, in which students had more opportunities of performing their translanguaging behavior. Students completing their collaborative learning activities were frequently seen making meaning/sense of negotiating meaning through translanguaging. It is not surprising that translanguaging interaction occurred in complicated activities that permitted all the resources kept in their repertoires.

### Students’ Translanguaging Practices

The following illustrates how the students embraced and incorporated translanguage strategies into the EFL classrooms. First, students’ translanguaging most frequently occurred when they clarified or negotiated information discussed in pairs and groups, and students seldom translanguage to affirm or reject information. Second, students were translanguage to comprehend and express information more clearly while assisting their peers who had to handle the information shared *via* translating it into the most accessible language.

96% of the students mentioned they “always” turn to bi/trilingual dictionaries, while 90% of the teachers noted the learners’ constant use of bi/trilingual dictionaries. Most students engage in bi/trilingual dictionaries when English meanings in English are not precise. A group of the students sheds some light on this translanguaging phenomenon. Here is an exemplar:

In EFL classes, when our teacher asked a question in English on an interactive whiteboard, we [peers with similar family languages] would translate what we had already known into Uyghur as well as Chinese with the aid of an electric bilingual dictionary at hand, and then answered our teacher in English. The topic seemed more evident, more explicit in mind using a language that we are pretty familiar with.

The EFL pedagogical features could range from translanguaging strategies to authentic teaching materials entirely in English in a multimedia classroom. This is especially true of NXC EFL students who tend to depend on their L1 or L2 to understand the L3 due to inadequate linguistic proficiency in dealing with content appropriate for their cognitive levels. In NXC EFL classes, it is contested that only the target language is used in class. Ironically, using L1 or L2 also facilitates L3 learning and helps reduce stress and anxiety among certain students.

When peers were meeting difficulties in exploring the meaning of a word, phrase, or sentence, they used Uyghur, Chinese, and English shifting ways.

The teacher moves from desk to desk when the students are learning collectively. The teacher's inspection aims to check the students' progress. The teacher is noticed to stop and squat to the students, … he would encourage the other peer members to explain in a familiar language (Uyghurs are usually urged to speak Uyghur if they were town persons). If they were not town persons, they were encouraged to talk to Chinese to handle their meeting issues. Code-switching, code-mixing, and translation are witnessed now and are very conducive to deepening their understanding of the lesson content, learning a new word well, and grasping the core meaning of a grammatical item.

Somewhat up to the teacher to decide about using learners’ linguistic resources students would speak other languages in EFL classes. Through peers’ translanguaging practices, a mutually supportive relationship could be established, beneficial for collaborative learning. The scaffolding function of translanguaging plays a role in peers’ establishing links between their different language resources (L1, L2, … Ln), which has been well acknowledged (Duarte, 2020). Translanguaging can provide this type of mutually interdependent scaffolding: gaining knowledge; growing in confidence.

### Cognition

The final affordance was cognition. From the translingual lens, learning a new language instructs the learners to become multilinguals through full use of all elements of their cognitive abilities with their previous experiences and knowledge. In the translanguaging pedagogical process, the learners’ linguistic repertoires should be expanded instead of emphasizing the English nativism standard. The teacher managed to create a sense of flexibility in the classroom.

I can sometimes be aware of my English teacher's shift in using different languages in class, but on most occasions, I have been engaged in learning without caring about the specific language I employed. My translanguaging awareness is developed and fostered by comparing and contrasting different named languages. The comparison and contrast activities were created to explore the similarities and discrepancies between phonetic, lexical, and semantic languages. These activities were customarily organized in a playful way to develop a sensory perception toward revealing the full potential of the other languages instead of treating students' other languages as impediments to learning a new language.

This also shows that the students were engaged in classroom-based activities and discussions around a given topic and took an active part in dynamic meaning-negotiating/making *via* engaging in multilingual languaging in a series of classroom activities. EFL teachers managed to direct the students’ attention to classroom discourse in completing the activities by coping with the students’ actual organic linguistic demands. They simultaneously activated the learners’ linguistic resources and provided translanguaging opportunities to develop multilingual competence instead of committing the monolingual bias (Blommaert, 2013) of merely using target language learning. In other words, their multilingualism was supported in their daily classes by balancing different linguistic codes the learners have had.

## Discussion

To develop translanguaging in EFL classrooms, we have identified that a translanguaging pedagogical framework is needed for its full implementation.

### The Necessity of Establishing a Translanguaging Pedagogical Framework

Drawn upon the data of this study, we find the initial translanguaging pedagogical framework for planning language alternation, which has been summarized by Lewis et al. (2012), who claimed “the planned alternation of the languages of input, processing, and output in a learning cycle.” When applying a translanguaging lens, teachers have to inquire whether the lesson content needs to be inaccessible for students as a prerequisite. If the possible answer is positive, teachers need to determine what teaching procedures should leverage students’ linguistic resources to ensure all students’ complete understanding of lesson content, demonstrate their learning outcomes, and reveal the questions they still have.

From a translanguaging lens, the students involved in an NXC EFL multimedia classroom have a communicative repertoire of scaffolding tools for learning, from which they select resources to communicate (Creese and Hornberger, 2008), so EFL instructors ought to be tolerant when allowing students to use L1 or L2 as needed. Their communicative repertoire is not a fixed set of sources for the students and is not the same at all times and spaces. To be more specific, translanguaging enables the flow of conversation in which pairs/groups of the students are collaborating, exploring more complex topics than they would if they used a target language only, as well as ensuring a shared understanding of an idea in a different language to explain, elaborate, or emphasize a point, or to repair comprehension difficulties with the assistance of multimedia facilities. Inevitably, translanguaging also offers teachers opportunities to engage students, resulting in better understanding (Yilmaz, 2021).

### Translanguaging as an Issue, a Natural Process, or a Resource

This research invited us to relook at the differences in language ideologies, which is conducive to understanding the necessity and significance of shifting from monolingual ideology to multilingual ideology. Up to now, we have identified a translanguaging continuum with two ends: one end is linked with the monolingual ideology, which treats translanguaging pedagogy as an issue of learning a new language; the other end is closely correlated with the multilingual ideology, which endorses translanguaging pedagogy as a resource for learning a new language. Between the two ends, across the continuum, models range from prioritizing the translanguaging pedagogy to denying the translanguaging as a vehicle to benefit the latest language learning. Teaching models are somewhere in the middle of the continuum, partially supporting the translanguaging pedagogy. The ideologies ranging from one end to another refer to intermingling, to a lesser or greater extent, the translanguaging pedagogy.

According to this continuum, we argue that the models of translanguaging pedagogy emphasize the concept of shared responsibility between monolingual ideology and multilingual ideology. For example, translanguaging solid pedagogy and students’ multimodal linguistic resources are of greater importance than teaching a new language through the new language. The most salient example of a solid translanguaging pedagogy is the NXC EFL classes, where the students’ entire repertoires have been leveraged in an orchestrated way. In a weak translanguaging pedagogy, the students’ reporters are not treated as valuable vehicles for furthering the aims of the new language learning. The range between the extremes lies in various models that draw on one or more resources to develop translanguaging competence. These models are only meant to serve as prototypes and adapted to fit the particular demands of the bi/multilingual learners and contexts. There is no translanguaging pedagogical template that is good for all. The models can be tailored to fit the learners’ particular communication needs. So translanguaging pedagogy can be viewed as an umbrella term for approaches that combine monolingual ideology and multilingual ideology, even if there are differences in the emphasis on the two doctrines.

### Pedagogical Implications

The present study also brings about some pedagogical implications: firstly, EFL teachers should consider that making sense of translanguaging as theory and pedagogy means teachers should pay special attention to adopting translanguaging positionality. If the prerequisite makes sense, the strategic translanguaging pedagogy will act more efficiently and effectively for bi/multilinguals in different stages of bi/multilingualism as dynamic agents in the discursive way of translanguaging with their entire linguistic repertoire (Yilmaz, 2021). Secondly, the translanguaging features transcend the boundaries of linguistic codes and modes, which are conducive to counteracting linguist inequities by giving equal voices to minoritized learners and displaying their unique identities in the classroom (Spotti et al., 2019; Yilmaz, 2021), *via* challenging the hegemony of English (García and Li, 2014; Yilmaz, 2021) and empowering learners in the classrooms (Yilmaz, 2021). Of course, there is no single approach to bilinguals, but what successful strategies have in common is that they illustrate an understanding of the underlying principles of bilingual education and are applied and evaluated appropriately in their particular contexts. Finally, we make a humble acknowledgment that through this study, the points raised and challenges overcome may be of interest to reforming bi/multilingual’s new language education, focusing on seeking effective and transformative ways to introduce new ideas in EFL pedagogy through a translanguaging lens.

## Conclusion

This research concludes that in the multilingual turn of EFL pedagogy, it is necessary to reconceptualize EFL pedagogy for multilingual students. Indeed, what best translanguaging space should be established and what ideal strategic translanguaging techniques should be adopted depends on a particular EFL context as a vital resource. Beyond this, students’ multilingual, multimodal and multi-semiotic resources should be availably valued as the most potent communicative means without hesitation. For this, teachers’ ideology toward multilingualism plays a pivotal role. If EFL educators stick to multilingualism as a natural phenomenon, they will offer informed considerations to all translanguaging strategies that can genuinely help the EFL teaching move out of English-only instructive ideology. It is hoped that this study can be one practical example of a multilingual turn in the EFL domain, where students’ needs can be thoughtfully put on the table and eventually satisfied. Overall, this research suggests an optimistic perspective on the future of EFL pedagogy.

## Data Availability Statement

The original contributions presented in the study are included in the article/supplementary material; further inquiries can be directed to the corresponding author.

## Ethics Statement

Ethical review and approval was not required for the study on human participants in accordance with the local legislation and institutional requirements. Written informed consent from the patients/participants or patients/participants legal guardian/next of kin was not required to participate in this study in accordance with the national legislation and the institutional requirements.

## Author Contributions

The author confirms being the sole contributor of this work and has approved it for publication.

## Funding

The work was supported by National Office for Philosophy and Social Sciences (17BYY104).

## Conflict of Interest

The author declares that the research was conducted in the absence of any commercial or financial relationships that could be construed as a potential conflict of interest.

## Publisher’s Note

All claims expressed in this article are solely those of the authors and do not necessarily represent those of their affiliated organizations, or those of the publisher, the editors and the reviewers. Any product that may be evaluated in this article, or claim that may be made by its manufacturer, is not guaranteed or endorsed by the publisher.
